# Impact of Sacbrood Virus on Larval Microbiome of *Apis mellifera* and *Apis cerana*

**DOI:** 10.3390/insects11070439

**Published:** 2020-07-13

**Authors:** Rujipas Yongsawas, Veeranan Chaimanee, Jeffery S. Pettis, Humberto Freire Boncristiani Junior, Dawn Lopez, Ammarin In-on, Panuwan Chantawannakul, Terd Disayathanoowat

**Affiliations:** 1 Department of Biology, Faculty of Science, Chiang Mai University, Chiang Mai 50200, Thailand; r.yongsawas@gmail.com (R.Y.); Panuwan@gmail.com (P.C.); 2 Department of Agro-Industrial Biotechnology, Maejo University Phrae Campus, Rong Kwang, Phrae 54140, Thailand; 3 Pettis and Associates LLC, Salisbury, MD 21801, USA; Pettis.jeff@gmail.com; 4 Honey Bee Research and Extension Laboratory, Entomology and Nematology Department, University of Florida, Gainnesville, FL 32611, USA; humbfb@gmail.com; 5 Bee Research Laboratory, USDA-ARS, Beltsville, MD 20705, USA; Dawn.Lopez@ARS.USDA.GOV; 6 Bioinformatics & Systems Biology Program, King Mongkut’s University of Technology Thonburi (Bang Khun Thian Campus), Bang Khun Thian, Bangkok 10150, Thailand; ammarin.ammarinin@mail.kmutt.ac.th; 7 Research Center in Bioresources for Agriculture, Industry and Medicine, Chiang Mai University, Chiang Mai 50200, Thailand; 8 Research Center of Microbial Diversity and Sustainable Utilization, Chiang Mai University, Chiang Mai 50200, Thailand

**Keywords:** *Apis mellifera*, *Apis cerana*, bee virus, Sacbrood virus, cross-infection, gut microbiome

## Abstract

In this study, we examined the impact of Sacbrood virus (SBV), the cause of larval honeybee (*Apis mellifera*) death, producing a liquefied a larva sac, on the gut bacterial communities on two larval honeybee species, *Apis mellifera* and *Apis cerana*. SBV was added into a worker jelly food mixture and bee larvae were grafted into each of the treatment groups for 24 h before DNA/RNA extraction. Confirmation of SBV infection was achieved using quantitative reverse transcription polymerase chain reaction (RT-qPCR) and visual symptomology. The 16S rDNA was sequenced by Illumina sequencing. The results showed the larvae were infected with SBV. The gut communities of infected *A. cerana* larvae exhibited a dramatic change compared with *A. mellifera*. In *A. mellifera* larvae, the Illumina sequencing revealed the proportion of *Gilliamella*, *Snodgrassella* and *Fructobacillus* was not significantly different, whereas in *A. cerana*, *Gilliamella* was significantly decreased (from 35.54% to 2.96%), however, with significant increase in *Snodgrassella* and *Fructobacillus*. The possibility of cross-infection should be further investigated.

## 1. Introduction

The Western cavity nesting bee *Apis mellifera* and Asiatic cavity nesting bee *Apis cerana* are two domesticated bee species in worldwide apiculture [1,2]. Sacbrood virus (SBV), a common single-stranded RNA virus belonging to the genus *Iflavirus* in the family *Iflaviridae* [3], was the first virus identified in honeybees [4]. The virus infects bee larvae, causing the larval development process to fail, leading to eventual death [5]. The infected larva changes color from white to yellow and eventually dark brown, drying out after death [6]. Additionally, the virus can infect adult bees showing no external symptoms [7], but surviving with a shorter life span [8,9]. SBV infection can be found in both *A. mellifera* and *A. cerana* [10,11,12,13], while the infection in *A. cerana* is more severe, in terms of prevalence and colony damage, than *A. mellifera* [14]. The consequences of this infection in *A. cerana* have been reported in Thailand, Vietnam, China, India and South Korea [5,15,16,17,18].

SBV can survive for up to four weeks in the larval remains of bees, pollen or honey [19]. The spread of the virus can occur in many ways, including the movement of honeycomb to other areas by a beekeeper, which spreads the virus to new locations. The natural swarming of bees may also carry the virus from a hive to new locations. Bee larvae (younger than eight days) can acquire the virus and exhibit symptoms, for example, from an adult bee collecting pollen that has an active viral infection contaminating hive food.

The gut microbiota of honeybees plays an important role in honeybee health. For example, the microbiota deals with processing an excess of carbohydrate and pectin lysate [20], which are important in supporting digestion in the honeybee gut. The bee-associated microbiota also has antimicrobial properties that provide potential inhibition of pathogens [21]. Furthermore, the gut microbiota is important in several aspects of host fitness and immunity. One example is the bacterial protein secretion system that improves protection against pathogens as well as bacterial nutrient production for host nutrient absorption [22].

The newly hatched larva has no, or minimal, gut bacteria until it receives its first feeding from a worker bee. Larvae are fed with royal jelly, honey, nectar or pollen, all which contain bacteria present in hive material. They will also acquire bacteria from the natural environment or the pollen that often contains bacteria, as the larvae develop into adult bees and assume foraging roles [23,24,25]. In addition to the microbiome, the health status of larval honeybees is further compounded by the use of pesticides, parasitic mites and outbreaks of bacterial and viral diseases [26].

We hypothesize that infection of SBV may result in dysbiosis of the bacterial communities in the honeybee larvae. The results reveal an overwhelming change in the bacterial microbiota in both species of honeybee larvae, *A. mellifera* and *A. cerana*, during viral infection, which has not been previously shown and has potential applications in apiculture worldwide.

## 2. Materials and Methods

### 2.1. Virus Inoculum

SBV was isolated from a symptomatic colony of *A. mellifera* larvae from the USDA honeybee research laboratory in Beltsville, Maryland. A total of 15 visually confirmed infected larvae, collected in a 50 mL tube containing 15 mL of phosphate-buffered saline (PBS), were homogenized and centrifuged at 2000× *g*. The supernatant was filtered using a 0.2 micron filter [27]. RNA was extracted from 200 µL of this preparation using TRIzol^TM^ Reagent (Thermo Fisher Scientific, Waltham, MA, USA) following the manufacturer’s protocols. To evaluate the levels of contaminating Deformed wing virus (DWV) (the most ubiquitous virus) in our SBV inoculum, we assessed both the SBV and DWV titers. RT-qPCR confirmed high titers of SBV and relatively lower titers of DWV in the SBV-symptomatic larvae. To eliminate the contamination of DWV (and other less prevalent viruses) found in the original sample, we performed serial dilutions using PBS and retested the sample until DWV was no longer found in the sample [27]. Primers for SBV and DWV and qPCR cycling parameters can be found in reference [28].

### 2.2. SBV Infection in Honeybee Larvae

Fifth instar larvae were collected from three different colonies each for *A. mellifera* and *A. cerana*. Larval age was determined by size in comparison to 1–4th instars. The colonies for both species were located in Phrae, Thailand and observed to be free of SBV symptoms. Ten larvae from each of the 6 colonies were grafted using a small grafting tool and placed onto a petri dish provisioned with a worker food mixture (6% D-glucose, 6% D-fructose, 1% yeast extract and 50% royal jelly) [29] spiked with high virus titer (10^6^ genome copies/larva), or no virus spike for a negative control consisting of uninoculated worker food. To assess viral infection and subsequent microbial activity, six larvae were removed from each test dish after 24 h of exposure and placed into 1.5 mL microcentrifuge with TRIzol^TM^ Reagent for RNA and DNA extraction.

### 2.3. Total RNA and DNA Extraction

The larval sample stored in TRIzol^TM^ Reagent (Thermo Fisher Scientific, Waltham, MA, USA) was extracted following the manufacturer’s protocol. The two phases were extracted separately and the colorless upper aqueous phase was transferred for extraction of total RNA. The interphase of the separated phase was processed for DNA extraction using the DNeasy Blood&Tissue Kit (Qiagen, Hilden, Germany). Total RNA and DNA were quantified and qualified using a Micro-Spectrophotometer NANO-200 (Allsheng, Hangzhou Allsheng Inc., Zhejiang, China).

### 2.4. SBV Expression in Honeybee Larvae

cDNA was prepared from 5000 ng RNA using the Tetro cDNA Synthesis Kit (Bioline USA Inc., Taunton, MA, USA) following manufacturers’ protocol. This cDNA was used as a template in quantitative real-time PCR on a Bio-Rad iCycler Thermal Cycler iQ5. Each reaction consisted of 250 ng cDNA with the SensiFAST^TM^ SYBR^®^ No-ROX Kit (Bioline USA Inc., MA, USA). Primers for qPCR were: Sacbrood virus (F: 5′-GCTCTAACCTCGCATCAAC-3′; R: 5′-TTGGAACTACGCATTCTCTG-3′), beta actin (F: 5′-TTGTATGCCAACACTGTCCTTT-3′; R: 5´-TGGCGCGATGATCTTAATTT-3′) and RpS5 (F: 5′-AATTATTTGGTCGCTGGAATTG-3′; R: 5′-TAACGTCCAGCAGAATGTGGTA-3′). The cycling parameters were 95 °C for 10 min, followed by 40 cycles of 95 °C 15 s and 60 °C 1 min, then holding at 4 °C. The relative gene expression data of SBV were analyzed using the 2^−ΔΔC^_T_ method [30]. The end products from qPCR (SBV, beta actin and RpS5) were purified and directly keep in −20 °C, then, sent for Sanger sequencing by Macrogen (Macrogen Inc., Seoul, Korea). All sequnecs were alignment by Basic Local Alignment Search Tool (BLAST—NCBI) and presented in Tables S1–S5.

The phylogenetics tree of SBV was constructed by Maximum Likelihood method and present in Figures S1 and S2.

### 2.5. Amplicon Sequencing Analysis

The extracted DNA had at least 2 ng/µL, purity value ≥1.7 and volume ≥30 µL. The qualified DNA was sent for next-generation sequencing for 16S amplicon (V3-V4 regions) using the Illumina platform with Illumina MiSeq model by Macrogen (Macrogen Inc., Seoul, Korea). The sequences were analyzed on Miseq SOP—Mothur program [31]. The Greengenes database version 13.8 was used to compare the bacterial sequences.

Rarefaction curves were generated using OTUs and Shannon index by PAST software version 3.14 [32].

### 2.6. Sequence Analysis

#### 2.6.1. Sequences Processing

Sequences were filtered for length, 0–469 base pairs, and reduced redundancy. A redundancy- reduced sequence was aligned to the Greengenes database version 13.8 as a reference and any poorly aligned sequences were removed. Chimera sequences were removed, along with any out of the ordinary sequences, before performing preliminary clustering of sequences and removing non-bacterial sequences by comparison to the Ribosomal Database Project (RDP) database.

#### 2.6.2. Clustering Sequences into Operational Taxonomic Units (OTUs)

A pairwise distance between sequences was calculated, then sequences were grouped into OTUs based on the OptiClust algorithm and each OTU was assigned a name.

### 2.7. Alpha Diversity Analysis

Comparison of gut microbial diversities was performed using PAST version 3.14 and indexed by the Simpson index.

### 2.8. Beta Diversity Analysis

The non-metric multidimensional scaling (NMDS) were used to determine gut bacterial community difference between healthy and infected honeybees. To perform significance tests of beta diversity, One-way PERMANOVA was used with the Bray–Curtis similarity index.

### 2.9. Statistical Analysis

Differences between samples were considered significant when *p* < 0.05. All statistical analyses were performed using PAST version 3.14. The differences in gut communities of honeybees between the healthy and infected were carried out with an unpaired t-test with Welch’s correction.

Linear discriminant analysis Effect Size (LEfSe) was used to determine the taxonomy of bacterial communities using the Galaxy application (http://huttenhower.sph.harvard.edu/galaxy) with a pairwise Wilcoxon test (<0.05) and Kruskal–Wallis test. The linear discriminant analysis (LDA) threshold score of bacteria was 2.0.

The Phylogenetic Investigation of Communities by Reconstruction of Unobserved States (PICRUSt) 1.0.0 (http://picrust.github.io/picrust) based on the Kyoto Encyclopedia of Genes and Genomes (KEGG) database was applied to group the bacterial communities and predict the functional genes [33,34,35].

## 3. Results

### 3.1. Sacbrood Virus Infection

**Figure 1:**
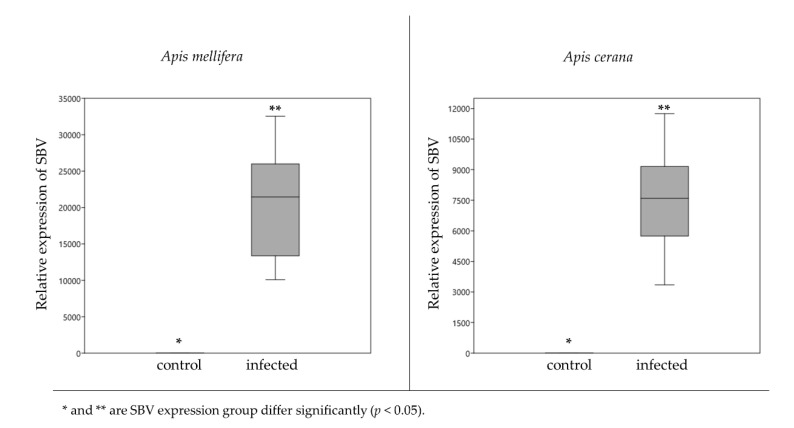
Relative expression of Sacbrood virus (SBV) boxplot showing the level of SBV detection in the control bees and infected larvae. SBV in the infected larvae is detected at significantly higher levels than in the control larvae, for both *A. mellifera* (t = 6.40, df = 10, *p* = 0.001) and *A. cerana* (t = 6.77, df = 10, *p* = 0.001).

The SBV relative expression indicating the level of infection in six bee larvae, for each treatment, was significantly higher than that in the control larvae of both *A. mellifera* (*p* = 0.001) and *A. cerana* (*p* = 0.001) (Figure 1). In *A. mellifera*, the relative expression of SBV in the control and infected larvae were 3.44 ± 0.7 and 20,635 ± 3221.03, respectively. For *A. cerana*, the SBV expression values were 2.62 ± 0.93 in the control larvae and 7520.45 ± 1110.65 in the infected larvae.

### 3.2. Larval Microbiome

#### 3.2.1. The Gut Bacteria of *A. mellifera* in the Control and Infected Larvae

**Figure 2:**
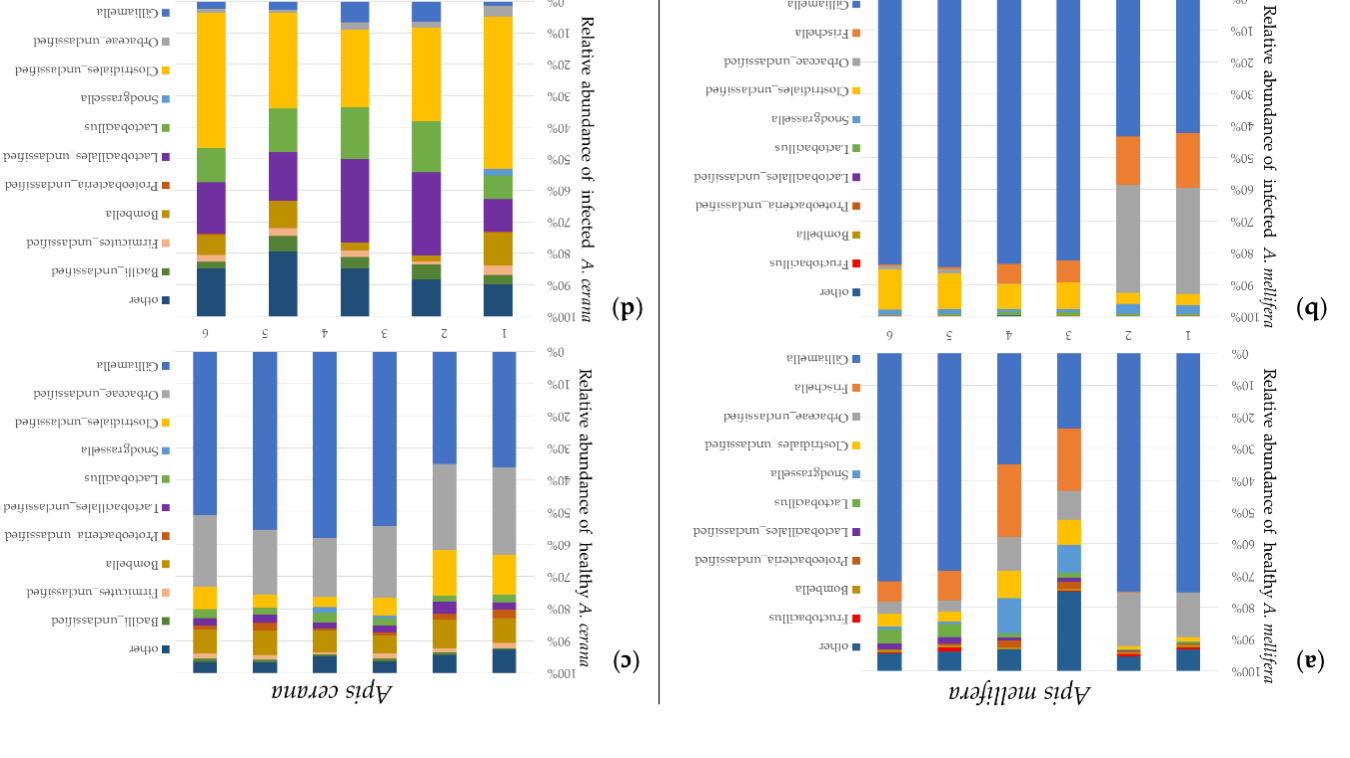
Proportions of bacterial communities found in control bees (**a**,**c**) and SBV-infected larval bees (**b**,**d**) of *A. mellifera* (**a**,**b**) and *A. cerana* (**c**,**d**).

The major identifiable gut bacterial component found in *A. mellifera* larvae was *Gilliamella* spp. (*p* = 0.006), *Lactobacillus* spp. (*p* = 0.012), *Bombella* spp. (*p* = 0.005) and *Fructobacillus* spp. (*p* = 0.187) (Figure 2a,b; Tables S6 and S7).

Proteobacteria showed the highest proportion of larval microbiome in both control (84.99%) and infected larvae (91.46%) of *A. mellifera* followed by Firmicutes with the proportions of 11.22% in control larvae and 8.46% in infected larvae. Comparison between both honeybee species showed that there was a significant difference in proportions of Proteobacteria and Firmicutes between the control and the infected larvae (Figure S3).

The percentage of major bacterial genera in control larvae of *A. mellifera* consisted of *Gilliamella* spp. (33.96%), *Frischella* spp. (9.69%), *Fructobacillus* spp. (4.85%), *Snodgrassella* spp. (2.85%) and *Lactobacillus* spp. (1.55%). For infected larvae of *A. mellifera*, this consisted of *Gilliamella* spp. (38.89%), *Frischella* spp. (9.70%) and *Snodgrassella* spp. (1.21%), while the proportion of *Fructobacillus* spp. and *Lactobacillus* spp. were decreased to 0.08% and 0.26%, respectively.

#### 3.2.2. The Larval Microbiome of *A. cerana* in the Control and Infected Larvae

The major identifiable larval microbiome component found in *A. cerana* was *Gilliamella* spp. (*p* = 0.0012), followed by *Bombella* spp. (*p* = 0.0044), *Lactobacillus* spp. (*p* = 0.0065) and *Snodgrassella* spp. (*p* = 0.5) (Figure 2c,d; Tables S8 and S9).

The larval microbiome percentage in the control larvae of *A. cerana* consisted of Proteobacteria (80.96%) followed by Firmicutes (15.34%). For the infected, we found that the Proteobacteria (17.76%) was the highest proportion in the infected larvae followed by Firmicutes (76.15%). There was a significant difference in Proteobacteria and Firmicutes proportions between the control and the infected larvae.

The proportion of major bacterial genera in the control larvae of *A. cerana* was *Gilliamella* spp. (35.54%), *Frischella* spp. (15.28%), *Fructobacillus* spp. (2.59%), *Snodgrassella* spp. (2.45%), *Lactobacillus* spp. (2.00%) and *Bombella* spp. (0.61%). In infected larvae of *A. cerana*, these were *Gilliamella* spp. (2.96%), *Frischella* spp. (18.60%), *Fructobacillus* spp. (7.49%), *Snodgrassella* spp. (15.99%), *Lactobacillus* spp. (6.54%) and *Bombella* spp. (2.06%).

### 3.3. Alpha Diversity Indices of Gut Bacterial Communities

**Figure 3:**
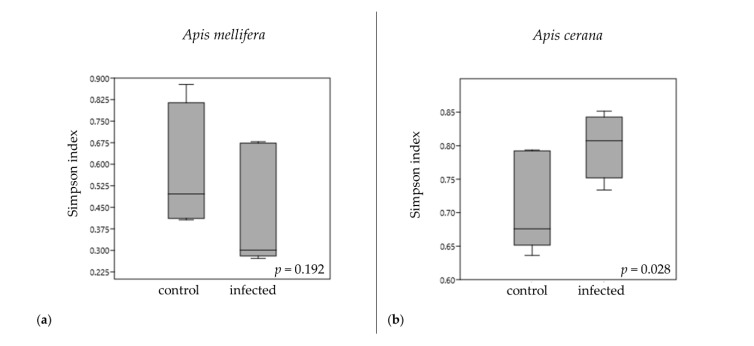
Alpha diversity boxplots compare the larval microbiome between the control and the infected worker larvae of *A. mellifera* (**a**) and *A. cerana* (**b**) designated by the Simpson index.

Alpha diversity was calculated with Simpson parameters (Figure 3) using PAST version 3.14 (Shannon and Chao 1 parameters were showed in Figure S4). For the larval microbiome abundance between the control and the infected groups in *A. mellifera*, the *p*-values of the diversity indexed by the Simpson index was 0.192 (Figure 3a). In *A. cerana*, the *p*-values of the diversity indexed by the Simpson index was 0.028 (Figure 3b). The rarefaction plot between OTU and the Shannon index is shown in Figure S5.

### 3.4. Beta Diversity Analysis of Larval Bacterial Communities

**Figure 4:**
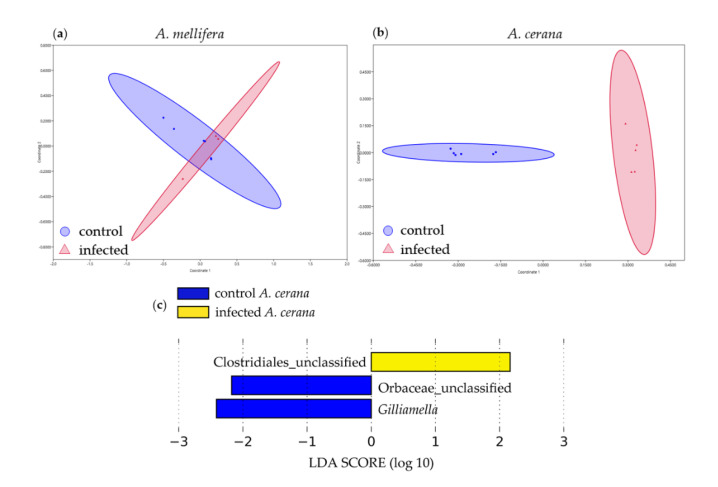
The non-metric multidimensional scaling (NMDS) analysis of larval bacterial communities in *A. mellifera* (**a**) and *A. cerana* (**b**) with linear discriminant analysis (LDA) score of bacteria that was affected by SBV infection (**c**).

The non-metric multidimensional scaling (NMDS) based on Bray–Curtis indices shows the composition of the gut bacterial communities of honeybees. The NMDS (Figure 4a,b) of *A. mellifera* and *A. cerana* were able to distinguish the gut bacteria flora between the control and SBV-infected larvae. The NMDS stress values of *A. mellifera* and *A. cerana* were 0.03 and 0.063, respectively.

The bacterial communities in infected groups in *A. cerana* were identified by LEfSe analysis and the differences were analyzed by one-way ANOVA. We found that unclassified Clostridiales (LDA = 2.55, *p* = 0.04) was affected by SBV infection, while the unclassified Orbaceae (LDA = 2.34, *p* = 5.53 × 10^−6^) and *Gilliamella* (LDA = 2.71, *p* = 0.0012) were abundantly present in the control group (Figure 4c; Table S10).

### 3.5. Functional Gene Prediction from 16S rRNA

**Figure 5:**
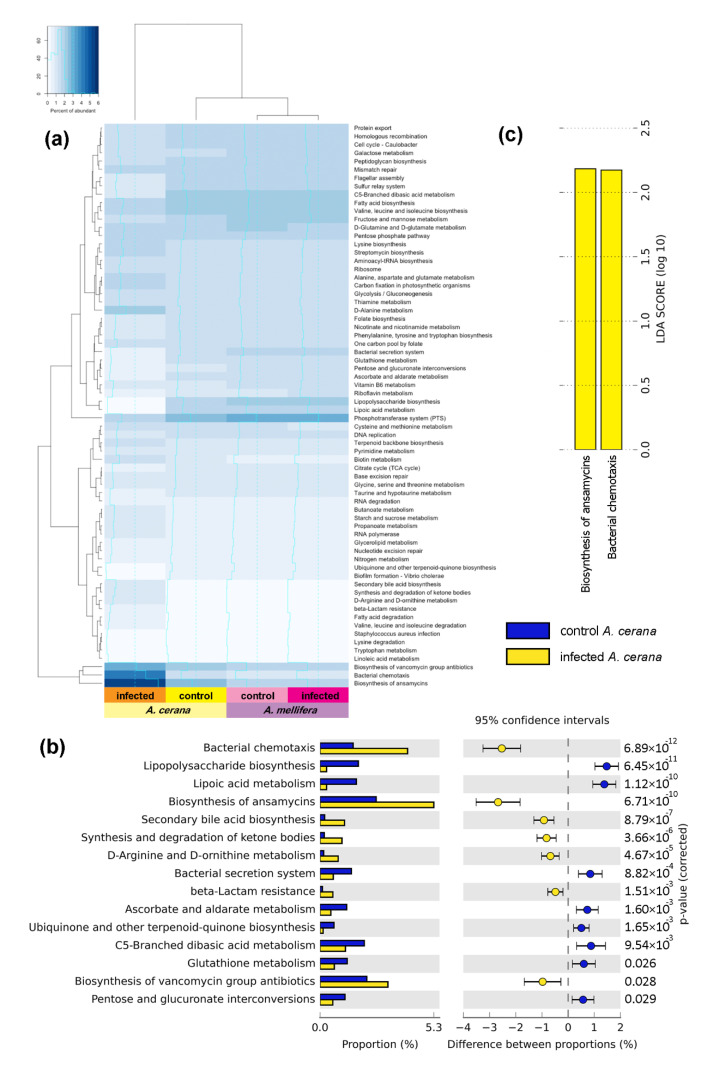
A heat map of gene expression in control and SBV-infected bee larvae. There was a high abundance of biosynthesis of ansamycins and bacterial chemotaxis function (**a**) with p-value of difference between proportions (**b**) and LDA score of biosynthesis of ansamycins and bacterial chemotaxis in bee larvae after viral infection (**c**).

The groups showing the most pronounced different functional genes between the control and SBV-infected bee larvae are shown on the heat map (Figure 5a). The functional groups’ biosyntheses of ansamycins (*p* = 6.71 × 10^−10^) and bacterial chemotaxis (*p* = 6.89 × 10^−12^) were found in *A. cerana* (Figure 5). The LDA score of these functions was more than 2.0 (Figure 5c), indicating that these genes were predominant after infection. The biosynthesis of ansamycins (LDA = 2.73), the natural antibacterial compounds against Gram-positive bacteria and bacterial chemotaxis (LDA = 2.64), and the movement of bacteria to higher concentration regions of beneficial chemical signal were mostly increased in SBV-infected larvae. Moreover, the biosynthesis of vancomycin group antibiotics (LDA = 2.51), secondary bile acid biosynthesis (LDA = 2.09), synthesis and degradation of ketone bodies (LDA = 2.07), D-arginine and D-ornithine metabolism (LDA = 1.98) and beta-lactam resistance (LDA = 1.82) was also elevated in infected larvae. On the contrary, the syntheses of C5-branched dibasic acid metabolism (LDA = 2.34), lipopolysaccharide biosynthesis (LDA = 2.22), lipoic acid metabolism (LDA = 2.21), bacterial secretion system (LDA = 2.18), glutathione metabolism (LDA = 2.12), ascorbate and aldarate metabolism (LDA = 2.12), pentose and glucuronate interconversions (LDA = 2.10), ubiquinone and other terpenoid-quinone biosynthesis (LDA = 1.80) were decreased in SBV-infected larvae (Table S11). 

## 4. Discussion

In this study, we investigated the interaction between viral infection and the larval microbiome using an NGS technique to compare the difference in gut microbial communities between control larvae and SBV-infected larvae, in two different species of honeybee, *A. mellifera* and *A. cerana*.

There are five species of core gut bacteria that dominated in this study in honeybees (*Apis* sp.) including *Gilliamella apicola*, *Snodgrassella alvi* [36], Lactobacillus Firm-4, Lactobacillus Firm-5 clade [37,38] and *Bifidobacterium asteroids* [39,40]. These bacteria are often found in adult worker bees [41]. Lactobacillus Firm-4 and Lactobacillus Firm-5 are the two most abundant species, followed by *G. apicola*, *S. alvi* and *B. asteroids*. Even though *B. asteroids* is less common in larvae, it is commonly found in adult worker bees [41].

In this study, *Gilliamella* spp. was the most abundant larval microbiome, and *Snodgrassella* spp. was found in smaller proportions, in both *A. mellifera* and *A. cerana*. It is possible that the use of bee larvae in this study was not equivocal to what occurs in adult bees. However, for the study of this viral brood disease, it is necessary. *Snodgrassella* spp., which is considered to be one of two major species in the honeybee microbiome, had a lower proportion of *Snodgrassella* in this study than previous studies. Lactobacillus detected in this study could only be grouped to the *Lactobacillus* spp. and unclassified Lactobacillus, as we were unable to identify the species. *Bifidobacterium* is found in small amounts in adult bees, therefore, the use of bee larvae in this study may account for the absence of *Bifidobacterium* in our results.

Cox-Foster et al. (2007) [42] report on a metagenomic survey indicating a high relative abundance of γ-proteobacteria in hives exhibiting symptoms of colony collapse disorder (CCD) and the disappearance phenomenon of worker bees from a colony, more so than in apparently healthy hives. This research reported the presence of Firmicutes and α-Proteobacteria (related to the genus Lactobacillus and acetic acid bacteria, respectively) was dramatically reduced in infected bees. In our study, the comparison between control and infected larvae of *Apis* spp., agrees with Cox-Foster et al. (2007) [42] in that *A. mellifera* larvae shows increased detection of γ-Proteobacteria taxa (*Gilliamella* and *Frischella*) and decreasing detection of Firmicutes (*Lactobacillus*). On the other hand, *A. cerana* larvae demonstrated an increase in Firmicutes (*Lactobacillus* and *Fructobacillus*), and a decrease in α-Proteobacteria (*Bombella*) and γ-Proteobacteria (*Gilliamella*), while *Frischella*, which belong to γ-Proteobacteria, still showed more abundance in the SBV-infected larvae.

We demonstrated dramatic changes in the larval microbiome of *A. cerana*, more so than in *A. mellifera* when infected with SBV. It is important to note that *A. mellifera* is the source of the SBV used in the inoculum for study. These results show that SBV isolated from *A. mellifera* is infective to *A. cerana* as well. Reddy et al. 2016 [43] described the genome of different SBV strains compared to Korean-SBV, which shares more than 90 percent similarity. *A. mellifera* may have experienced host adaptation to this virus, which could explain the less severe changes found in the larval microbiome compared to those seen in *A. cerana*.

In addition, research by Engel et al. (2012) [44] on genomic analysis of gut microbiota revealed some members of the gut microbiome in adult bees promoted increased quality of life of the host. This research showed high abundance of pectin-degrading enzyme genes in the gut metagenome of the adult honeybee. The extreme decrease in *Gilliamella* in this study in *A. cerana* larvae may reveal that the activity in pectin degradation comes from this bacterium, supporting the theory that *Gilliamella* helps decompose pectin for honeybees [44]. This could negatively impact *A. cerana* larval longevity during infection with this virus.

The functional gene analysis base on the KEGG database allowed for an analysis of the biological activities found in the bacterial communities of each bee larval sample group. The most abundant functional gene found in *A. cerana* larvae was ansamycins, the group of antibiotics which have a naphthalene ring. The antibiotics belonging to the ansamycins group are rifamycins, halomycins, tolypomycin Y, streptovaricins, naphthomycin [45], and geldanamycin [46]. Rifamycins, halomycins, tolypomycin Y and streptovaricins have biological activity against Gram-positive bacteria and *Mycobacterium tuberculosis* by inhibiting RNA synthesis [44]. Naphthomycin has reported cytotoxicity against A-549 and P388 cell lines, but inhibitory activity against Gram-positive bacteria and *M. tuberculosis* was not observed [47]. The ansamycins group mostly produced by actinomycetes, *Streptomyces* sp., has not yet been reported in the family of Clostridiales in the phylum Firmicutes. In this analysis, biosynthesis of ansamycins function was the most abundant in infected *A. cerana* larvae, which relates to increasing unclassified Clostridiales. This relation may explain that Clostridiales could be able to produce ansamycin antibiotics, but under which conditions has yet to be studied. However, actinomycetes was found in a small amount in infected *A. cerana* larvae, which did not affect the gut communities as determined by LEfSe analysis. Therefore, we suggest that actinomycetes are not related to the functional genes. Another of the most abundant functional genes was bacterial chemotaxis, the movement toward or away from chemicals [48], that could be found in many bacteria including Clostridiales. This could explain the increased movement of bacteria after viral infection in *A. cerana* larvae. When bee larva become infected, this could affect the activities of organs and change the chemical composition in larvae. Subsequently, gut bacteria may be attracted to the new chemical composition or alternatively, move away from toxic chemicals produced in infected larvae, leading to increase the bacterial chemotaxis activities in infected *A. cerana*. The advantage and/or disadvantage of this chemotaxis against Sacbrood virus needs to be further analyzed. Nevertheless, the 16s rRNA gene prediction in this study was analyzed from adult honeybees, of which *Gilliamella* (and Orbaceae) had an overwhelming versatility of gene function. We suggest that information on the larval microbiome, gained in future studies, might be helpful to explain their exact function. 

## 5. Conclusions

The *A. mellifera*-SBV used in this study led to a cross infection in *A. cerana* and caused significant changes in the larval microbiome, while in *A. mellifera*, less changes in the bacterial community were observed. It is possible that *A. mellifera* may have a host adaptation to this virus, or *A. mellifera*-SBV does not affect the bacterial community. On the contrary, in *A. cerana*, *A. mellifera*-SBV infection may or may not cause symptoms of infection, but the *A. mellifera*-SBV dramatically changed the larval microbiome in *A. cerana*. Therefore, it is imperative to separate *A. cerana*, in apiculture, from *A. mellifera* or other bees that pose the risk of being a Sacbrood virus carrier, passing a virus to an un-adapted host.

## Supplementary Materials

The following are available online at https://www.mdpi.com/2075-4450/11/7/439/s1, Figure S1: Phylogenetic tree of Sacbrood virus in infected honey bees. Figure S2: Bootstrap consensus tree of Sacbrood virus in infected honey bees. Figure S3: Proportion of gut bacterial community (phylum) found in healthy and infected honeybees. Figure S4: Alpha diversity boxplots compare the gut microbiota between the control and infected worker larvae of *A. mellifera* (a) and *A. cerana* (b) designated by Shannon and Chao 1 index. Figure S5: Rarefaction curves of bacterial OTUs and Shannon index constructed by PAST software version 3.14. Table S1. Sacbrood virus sequence alignment by Basic Local Alignment Search Tool (BLAST—NCBI), to confirm SBV, extracted from 3 sampled infected larvae of each *A. mellifera* and *A. cerana*. Table S2. Beta actin sequence alignment by Basic Local Alignment Search Tool (BLAST—NCBI), to confirm the beta actin gene of *A. cerana*. Table S3. Beta actin sequence alignment by Basic Local Alignment Search Tool (BLAST—NCBI), to confirm the beta actin gene of *A. mellifera*. Table S4. Ribosomal Protein S5 sequence alignment by Basic Local Alignment Search Tool (BLAST—NCBI), to confirm the ribosomal protein S5 gene of *A. cerana*. Table S5. Ribosomal protein S5 sequence alignment by Basic Local Alignment Search Tool (BLAST—NCBI), to confirm the ribosomal protein S5 gene of *A. mellifera*. Table S6: Proportion of bacterial genera found in control *A. mellifera*. Table S7: Proportion of bacterial genera found in SBV infected *A. mellifera*. Table S8: Proportion of bacterial genera found in control *A. cerana*. Table S9: Proportion of bacterial genera found in SBV infected *A. cerana*. Table S10. LDA score of Functional gene prediction based on KEGG database. Table S11. LDA score of bacteria affected in *A. cerana*.
