# Supplementary material for: Impact of Sacbrood Virus on Larval Microbiome of Apis mellifera and Apis cerana
Source: Insects. 2020 Jul 13;11(7):439. doi: 10.3390/insects11070439 (PMC7411915; doi:10.3390/insects11070439)
Supplement: Supplementary file 1 [file insects-11-00439-s001.pdf]

**Table S1.** Sacbrood virus sequence alignment by Basic Local Alignment Search Tool (BLAST - NCBI), to confirm SBV, extracted from 3 sampled infected larvae of each *A. mellifera* and *A. cerana*.

| Sample | Query Length | Matching | Max Score | Total Score | Query Cover | E value | Per. Ident | Accession |
|---|---|---|---|---|---|---|---|---|
| SBV_AC1 | 348 | Sacbrood virus isolate MD1, complete genome | 532 | 532 | 98% | 3.00E-147 | 95.06% | MG545286.1 |
| SBV_AC2 | 349 | Sacbrood virus isolate MD1, complete genome | 540 | 540 | 98% | 2.00E-149 | 95.36% | MG545286.1 |
| SBV_AC3 | 347 | Sacbrood virus isolate MD1, complete genome | 553 | 553 | 93% | 3.00E-153 | 97.54% | MG545286.1 |
| SBV_AM1 | 346 | Sacbrood virus isolate MD1, complete genome | 564 | 564 | 98% | 1.00E-156 | 96.77% | MG545286.1 |
| SBV_AM2 | 350 | Sacbrood virus isolate MD1, complete genome | 534 | 534 | 98% | 9.00E-148 | 95.09% | MG545286.1 |
| SBV_AM3 | 348 | Sacbrood virus isolate MD1, complete genome | 538 | 538 | 98% | 7.00E-149 | 95.35% | MG545286.1 |

AC is *A. cerana.*

AM is *A. mellifera.*

**Table S2.** Beta actin sequence alignment by Basic Local Alignment Search Tool (BLAST - NCBI), to confirm the beta actin gene of *A. cerana*.

| Sample | Query Length | Matching | Max Score | Total Score | Query Cover | E value | Per. Ident | Accession |
|---|---|---|---|---|---|---|---|---|
| AC1 | 125 | Apis cerana mRNA for actin, partial cds | 193 | 193 | 99% | 2.00E-45 | 95.16% | AB072495.1 |
| AC2 | 123 | Apis cerana mRNA for actin, partial cds | 213 | 213 | 99% | 1.00E-51 | 98.36% | AB072495.1 |
| AC3 | 127 | Apis cerana mRNA for actin, partial cds | 183 | 183 | 99% | 1.00E-42 | 93.65% | AB072495.1 |
| SBV_AC1 | 124 | Apis cerana mRNA for actin, partial cds | 204 | 204 | 99% | 9.00E-49 | 96.75% | AB072495.1 |
| SBV_AC2 | 127 | Apis cerana mRNA for actin, partial cds | 189 | 189 | 99% | 2.00E-44 | 94.44% | AB072495.1 |
| SBV_AC3 | 143 | Apis cerana mRNA for actin, partial cds | 198 | 198 | 86% | 5.00E-47 | 95.97% | AB072495.1 |

AC is *A. cerana.*

SBV_AC is infected *A. cerana.*

**Table S3.** Beta actin sequence alignment by Basic Local Alignment Search Tool (BLAST - NCBI), to confirm the beta actin gene of *A. mellifera*.

| Sample | Query Length | Matching | Max Score | Total Score | Query Cover | E value | Per. Ident | Accession |
|---|---|---|---|---|---|---|---|---|
| AM1 | 124 | Apis mellifera mRNA for actin, partial cds | 209 | 209 | 99% | 2.00E-50 | 97.56% | AB023025.1 |
| AM2 | 126 | Apis mellifera mRNA for actin, partial cds | 206 | 206 | 99% | 2.00E-49 | 96.80% | AB023025.1 |
| AM3 | 125 | Apis mellifera mRNA for actin, partial cds | 204 | 204 | 99% | 9.00E-49 | 96.77% | AB023025.1 |
| SBV_AM1 | 123 | Apis mellifera mRNA for actin, partial cds | 213 | 213 | 99% | 1.00E-51 | 98.36% | AB023025.1 |
| SBV_AM2 | 126 | Apis mellifera mRNA for actin, partial cds | 200 | 200 | 99% | 1.00E-47 | 96.00% | AB023025.1 |
| SBV_AM3 | 125 | Apis mellifera mRNA for actin, partial cds | 209 | 209 | 99% | 2.00E-50 | 97.58% | AB023025.1 |

AM is *A. mellifera.*

SBV_AM is infected *A. mellifera.*

**Table S4.** Ribosomal Protein S5 sequence alignment by Basic Local Alignment Search Tool (BLAST - NCBI), to confirm the ribosomal protein S5 gene of *A. cerana*.

| Sample | Accession | Query Length | Matching | Max Score | Total Score | Query Cover | E value | Per. Ident | Accession |
|---|---|---|---|---|---|---|---|---|---|
| AC1 | | 123 | PREDICTED: Apis cerana 40S ribosomal protein S5 (LOC107998344), transcript variant X2, mRNA | 189 | 189 | 96% | 2.00E-44 | 95.80% | XM_017057561.2 |
| AC2 | | 119 | PREDICTED: Apis cerana 40S ribosomal protein S5 (LOC107998344), transcript variant X2, mRNA | 202 | 202 | 96% | 3.00E-48 | 98.26% | XM_017057561.2 |
| AC3 | | 119 | PREDICTED: Apis cerana 40S ribosomal protein S5 (LOC107998344), transcript variant X2, mRNA | 196 | 196 | 96% | 1.00E-46 | 97.39% | XM_017057561.2 |
| SBV_AC1 | | 121 | PREDICTED: Apis cerana 40S ribosomal protein S5 (LOC107998344), transcript variant X2, mRNA | 193 | 193 | 96% | 2.00E-45 | 96.58% | XM_017057561.2 |
| SBV_AC2 | | 120 | PREDICTED: Apis cerana 40S ribosomal protein S5 (LOC107998344), transcript variant X2, mRNA | 187 | 187 | 98% | 8.00E-44 | 95.76% | XM_017057561.2 |

AC is *A. cerana.*

SBV_AC is infected *A. cerana.*

**Table S5.** Ribosomal protein S5 sequence alignment by Basic Local Alignment Search Tool (BLAST - NCBI), to confirm the ribosomal protein S5 gene of *A. mellifera*.

| Sample | Query Length | Matching | Max Score | Total Score | Query Cover | E value | Per. Ident | Accession |
|---|---|---|---|---|---|---|---|---|
| AM1 | 123 | PREDICTED: Apis mellifera 40S ribosomal protein S5 (LOC409728), transcript variant X2, mRNA | 198 | 198 | 95% | 4.00E-47 | 97.46% | XM_006570237.3 |
| AM2 | 119 | PREDICTED: Apis mellifera 40S ribosomal protein S5 (LOC409728), transcript variant X2, mRNA | 198 | 198 | 98% | 4.00E-47 | 97.44% | XM_006570237.3 |
| AM3 | 119 | PREDICTED: Apis mellifera 40S ribosomal protein S5 (LOC409728), transcript variant X2, mRNA | 213 | 213 | 96% | 1.00E-51 | 100.00% | XM_006570237.3 |
| SBV_AM1 | 122 | PREDICTED: Apis mellifera 40S ribosomal protein S5 (LOC409728), transcript variant X2, mRNA | 198 | 198 | 96% | 4.00E-47 | 97.46% | XM_006570237.3 |
| SBV_AM2 | 119 | PREDICTED: Apis mellifera 40S ribosomal protein S5 (LOC409728), transcript variant X2, mRNA | 213 | 213 | 96% | 1.00E-51 | 100.00% | XM_006570237.3 |
| SBV_AM3 | 120 | PREDICTED: Apis mellifera 40S ribosomal protein S5 (LOC409728), transcript variant X2, mRNA | 206 | 206 | 98% | 2.00E-49 | 98.31% | XM_006570237.3 |

AM is *A. mellifera.*

SBV_AM is infected *A. mellifera.*

**Table S6:** Proportion of bacterial genera found in control *A. mellifera*.

| Genus | proportion (%) | | | | | |
|---|---|---|---|---|---|---|
| | **healthy 1** | **healthy 2** | **healthy 3** | **healthy 4** | **healthy 5** | **healthy 6** |
| *Gilliamella* | 75.27 | 75.16 | 23.73 | 34.92 | 68.45 | 71.74 |
| *Frischella* | 0.00 | 0.16 | 19.48 | 22.86 | 9.23 | 6.52 |
| Orbaceae unclassified | 13.98 | 16.77 | 9.21 | 10.63 | 3.65 | 3.75 |
| Clostridiales unclassified | 1.38 | 1.11 | 7.91 | 8.57 | 3.00 | 3.95 |
| *Snodgrassella* | 0.15 | 0.16 | 8.85 | 11.11 | 0.86 | 0.99 |
| *Lactobacillus* | 0.46 | 0.32 | 1.42 | 1.43 | 4.08 | 4.35 |
| Lactobacillales unclassified | 0.15 | 0.16 | 1.30 | 0.79 | 2.15 | 1.98 |
| Proteobacteria unclassified | 0.31 | 0.32 | 2.13 | 2.22 | 0.43 | 0.00 |
| *Bombella* | 0.77 | 0.47 | 0.71 | 0.63 | 0.86 | 0.79 |
| *Fructobacillus* | 0.61 | 0.79 | 0.12 | 0.00 | 1.07 | 0.40 |
| other | 6.91 | 4.59 | 25.15 | 6.83 | 6.22 | 5.53 |

**Table S7:** Proportion of bacterial genera found in SBV infected *A. mellifera*.

| Genus | proportion (%) | | | | | |
|---|---|---|---|---|---|---|
| | **infected 1** | **infected 2** | **infected 3** | **infected 4** | **infected 5** | **infected 6** |
| *Gilliamella* | 42.31 | 43.32 | 82.38 | 83.51 | 84.54 | 83.66 |
| *Frischella* | 17.27 | 15.40 | 6.85 | 6.18 | 0.68 | 0.56 |
| Orbaceae unclassified | 33.28 | 33.81 | 0.10 | 0.10 | 1.18 | 1.01 |
| Clostridiales unclassified | 3.51 | 3.57 | 8.42 | 7.92 | 11.33 | 12.67 |
| *Snodgrassella* | 3.10 | 3.30 | 0.97 | 0.90 | 1.74 | 1.59 |
| *Lactobacillus* | 0.24 | 0.25 | 0.91 | 0.95 | 0.31 | 0.29 |
| Lactobacillales unclassified | 0.04 | 0.03 | 0.01 | 0.03 | 0.01 | 0.01 |
| Proteobacteria unclassified | 0.04 | 0.05 | 0.00 | 0.01 | 0.01 | 0.01 |
| *Bombella* | 0.05 | 0.07 | 0.20 | 0.20 | 0.13 | 0.09 |
| *Fructobacillus* | 0.07 | 0.08 | 0.00 | 0.00 | 0.00 | 0.01 |
| other | 0.10 | 0.11 | 0.16 | 0.19 | 0.08 | 0.12 |

**Table S8:** Proportion of bacterial genera found in control *A. cerana*.

| Genus | proportion (%) | | | | | |
|---|---|---|---|---|---|---|
| | **healthy 1** | **healthy 2** | **healthy 3** | **healthy 4** | **healthy 5** | **healthy 6** |
| *Gilliamella* | 36.17 | 34.93 | 54.30 | 58.13 | 55.43 | 50.83 |
| *Frischella* | 27.10 | 26.85 | 22.20 | 18.19 | 20.09 | 22.28 |
| Orbaceae unclassified | 12.28 | 14.11 | 5.61 | 3.27 | 4.22 | 7.14 |
| Clostridiales unclassified | 7.58 | 8.76 | 5.53 | 6.61 | 7.76 | 7.40 |
| *Snodgrassella* | 2.18 | 3.75 | 2.23 | 2.08 | 2.67 | 2.26 |
| *Lactobacillus* | 2.53 | 1.93 | 2.15 | 2.82 | 2.07 | 2.70 |
| Lactobacillales unclassified | 2.76 | 1.93 | 1.00 | 0.67 | 2.16 | 1.31 |
| Proteobacteria unclassified | 1.61 | 1.37 | 1.61 | 0.67 | 1.47 | 1.65 |
| *Bombella* | 0.57 | 0.80 | 0.84 | 0.59 | 0.78 | 1.04 |
| *Fructobacillus* | 0.00 | 0.00 | 0.92 | 1.78 | 0.00 | 0.00 |
| other | 7.23 | 5.57 | 3.61 | 5.20 | 3.36 | 3.39 |

**Table S9:** Proportion of bacterial genera found in SBV infected *A. cerana*.

| Genus | proportion (%) | | | | |
|---|---|---|---|---|---|
| | **infected 1** | **infected 2** | **infected 3** | **infected 4** | **infected 5** |
| *Gilliamella* | 1.45 | 6.44 | 6.64 | 2.58 | 2.39 |
| *Frischella* | 3.38 | 1.87 | 2.36 | 1.15 | 1.30 |
| Orbaceae unclassified | 48.39 | 29.73 | 24.63 | 30.37 | 42.83 |
| Clostridiales unclassified | 10.45 | 1.87 | 2.78 | 8.60 | 6.30 |
| *Snodgrassella* | 10.29 | 26.40 | 26.34 | 15.47 | 16.52 |
| *Lactobacillus* | 7.56 | 16.22 | 16.49 | 13.75 | 10.87 |
| Lactobacillales unclassified | 0.32 | 0.00 | 0.00 | 0.00 | 0.22 |
| Proteobacteria unclassified | 2.89 | 1.04 | 1.93 | 2.58 | 2.17 |
| *Bombella* | 3.05 | 4.78 | 3.64 | 4.87 | 2.17 |
| *Fructobacillus* | 2.09 | 0.00 | 0.00 | 0.00 | 0.00 |
| other | 10.13 | 11.64 | 15.20 | 20.63 | 15.22 |

**Table S10**. LDA score of Functional gene prediction based on KEGG database.

| Functional gene | LDA score | increase/decrease |
|---|---|---|
| Lipopolysaccharide biosynthesis | 2.22141423784 | decrease |
| Ascorbate and aldarate metabolism | 2.11809931208 | decrease |
| Ubiquinone and other terpenoid-quinone biosynthesis | 1.7923916895 | decrease |
| Pentose and glucuronate interconversions | 2.0960405543 | decrease |
| Lipoic acid metabolism | 2.21484384805 | decrease |
| Glutathione metabolism | 2.12139568071 | decrease |
| Bacterial secretion system | 2.17609125906 | decrease |
| C5-Branched dibasic acid metabolism | 2.33994806169 | decrease |
| D-Arginine and D-ornithine metabolism | 1.98000337158 | increase |
| Biosynthesis of vancomycin group antibiotics | 2.51121470114 | increase |
| beta-Lactam resistance | 1.81624129999 | increase |
| Secondary bile acid biosynthesis | 2.09342168516 | increase |
| Biosynthesis of ansamycins | 2.73359846096* | increase |
| Synthesis and degradation of ketone bodies | 2.07371835035 | increase |
| Bacterial chemotaxis | 2.63748972951* | increase |

* These are the most affected functions in SBV infected *A. cerana*

**Table S11**. LDA score of bacteria affected in *A. cerana*.

| Bacteria taxa | LDA score |
|---|---|
| *Gilliamella* | 2.70520346596** |
| *Lactobacillus* | 2.11316664674 |
| *Bombella* | 1.85797792939 |
| *Snodgrassella* | 0.73293969148 |
| Firmicutes_unclassified | 1.32688027974 |
| Bacilli_unclassified | 1.56870843394 |
| Clostridiales_unclassified | 2.54640904842* |
| Proteobacteria_unclassified | 1.14991384179 |
| Lactobacillales_unclassified | 2.27887045036 |
| Orbaceae_unclassified | 2.34084185769** |
| other | 2.16329390352 |

* This is the most affected function in SBV infected *A. cerana*

**These are the most affected functions in healthy *A. cerana*

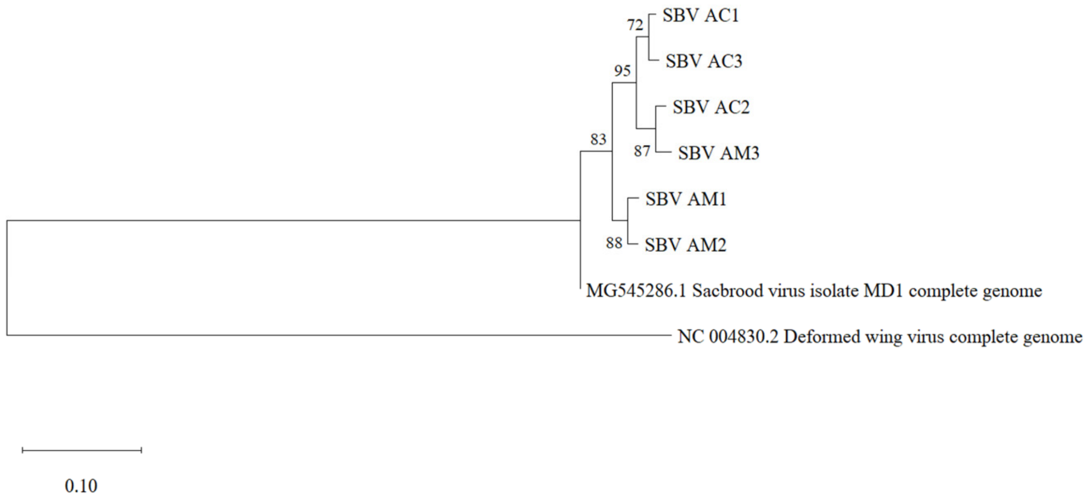


**Figure S1:** Phylogenetic tree of Sacbrood virus in infected honey bees.

**Evolutionary analysis by Maximum Likelihood method**

The evolutionary history was inferred by using the Maximum Likelihood method and Tamura 3-parameter model [1]. The tree with the highest log likelihood (-1250.85) is shown. The percentage of trees in which the associated taxa clustered together is shown next to the branches. Initial tree(s) for the heuristic search were obtained automatically by applying Neighbor-Join and BioNJ algorithms to a matrix of pairwise distances estimated using the Maximum Composite Likelihood (MCL) approach, and then selecting the topology with superior log likelihood value. The tree is drawn to scale, with branch lengths measured in the number of substitutions per site. This analysis involved 8 nucleotide sequences. There were a total of 371 positions in the final dataset. Evolutionary analyses were conducted in MEGA X [2].

1. Tamura K. (**1992**). Estimation of the number of nucleotide substitutions when there are strong transition-transversion and G + C-content biases. *Molecular Biology and Evolution* **9**:678-687.

2. Kumar S., Stecher G., Li M., Knyaz C., and Tamura K. (**2018**). MEGA X: Molecular Evolutionary Genetics Analysis across computing platforms. *Molecular Biology and Evolution* **35**:1547-1549.

3. Felsenstein J. (**1985**). Confidence limits on phylogenies: An approach using the bootstrap. *Evolution* **39**:783-791.

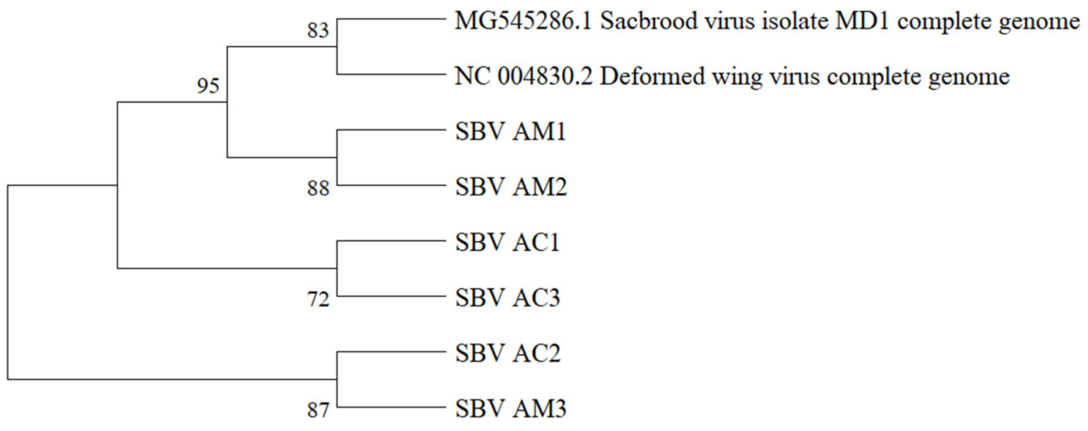


**Figure S2:** Bootstrap consensus tree of Sacbrood virus in infected honey bees.

**Evolutionary analysis by Maximum Likelihood method**

The evolutionary history was inferred by using the Maximum Likelihood method and Tamura 3-parameter model [1]. The bootstrap consensus tree inferred from 1000 replicates [3] is taken to represent the evolutionary history of the taxa analyzed [3]. Branches corresponding to partitions reproduced in less than 50% bootstrap replicates are collapsed. The percentage of replicate trees in which the associated taxa clustered together in the bootstrap test (1000 replicates) are shown next to the branches [3]. Initial tree(s) for the heuristic search were obtained automatically by applying Neighbor-Join and BioNJ algorithms to a matrix of pairwise distances estimated using the Maximum Composite Likelihood (MCL) approach, and then selecting the topology with superior log likelihood value. This analysis involved 8 nucleotide sequences. There were a total of 371 positions in the final dataset. Evolutionary analyses were conducted in MEGA X [2].

1. Tamura K. (**1992**). Estimation of the number of nucleotide substitutions when there are strong transition-transversion and G + C-content biases. *Molecular Biology and Evolution* **9**:678-687.

2. Kumar S., Stecher G., Li M., Knyaz C., and Tamura K. (**2018**). MEGA X: Molecular Evolutionary Genetics Analysis across computing platforms. *Molecular Biology and Evolution* **35**:1547-1549.

3. Felsenstein J. (**1985**). Confidence limits on phylogenies: An approach using the bootstrap. *Evolution* **39**:783-791.

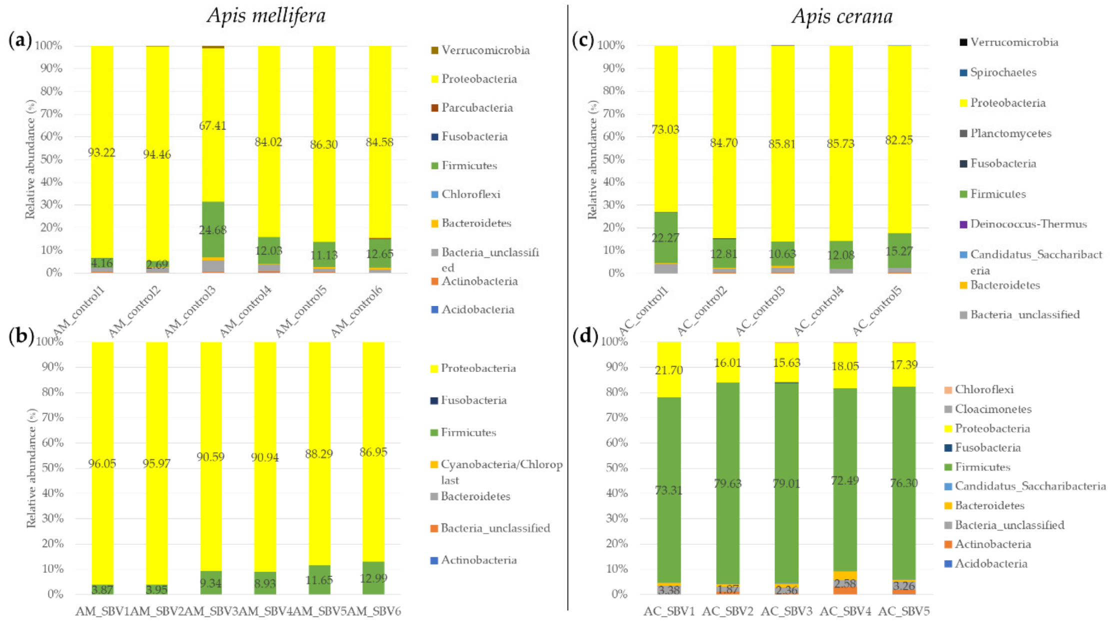


**Figure S3:** Proportion of gut bacterial community (phylum) found in healthy and infected honeybees.

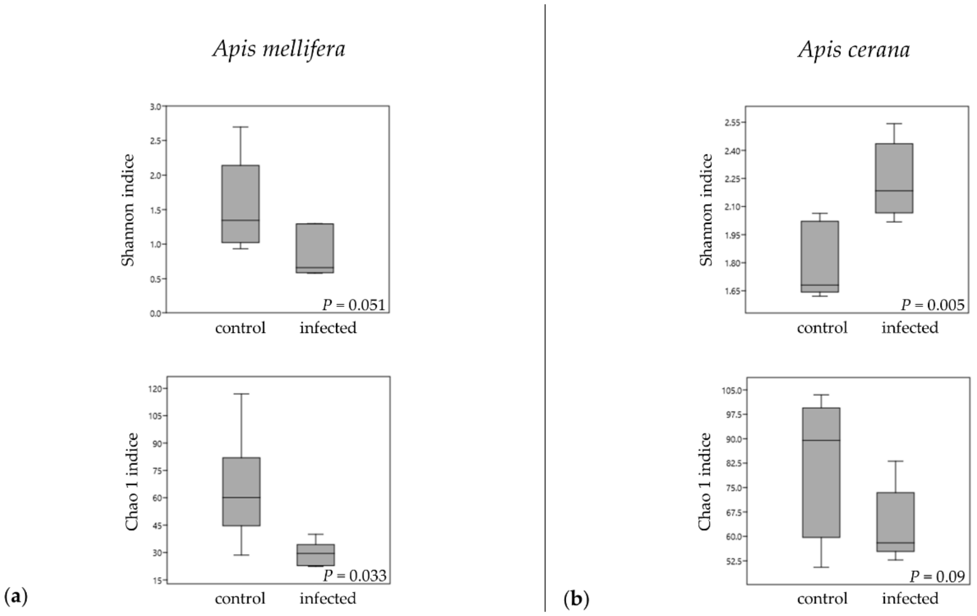


**Figure S4:** Alpha diversity boxplots compare the gut microbiota between the control and infected worker larvae of *A. mellifera* (a) and *A. cerana* (b) designated by Shannon and Chao 1 index.

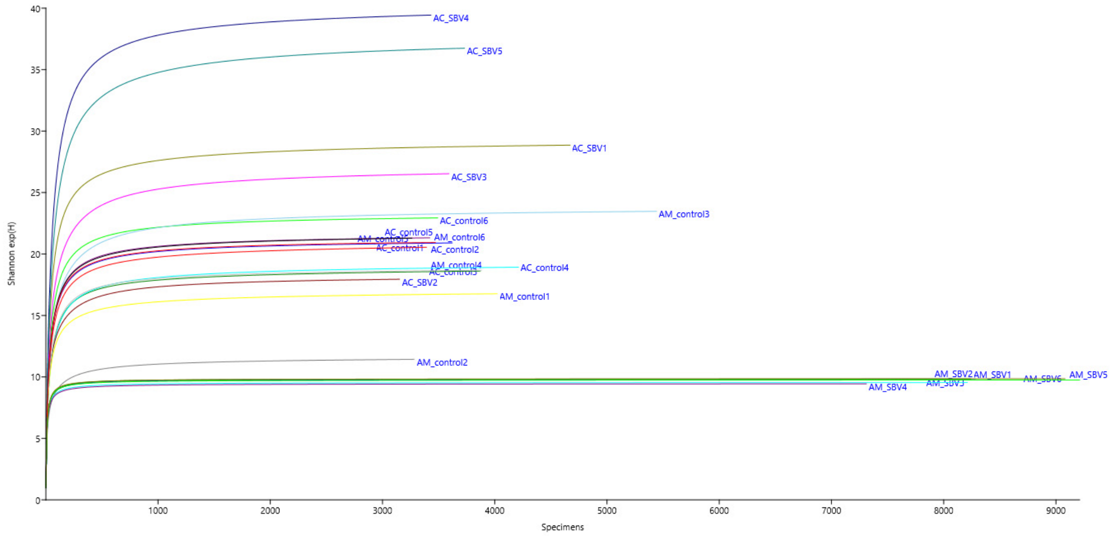


**Figure S5:** Rarefaction curves of bacterial OTUs and Shannon index constructed by PAST software version 3.14.
